# Supplementary figures and images for: A multi-group path analysis of medication documentation quality using cross-sectional survey data: Impact of leadership, job satisfaction, patient-related burnout, and patient safety culture
Source: PLoS One. 2025 Sep 12;20(9):e0330499. doi: 10.1371/journal.pone.0330499 (PMC12431215; doi:10.1371/journal.pone.0330499)

## Supplementary Materials

Figure S1: The model used in the path analysis.

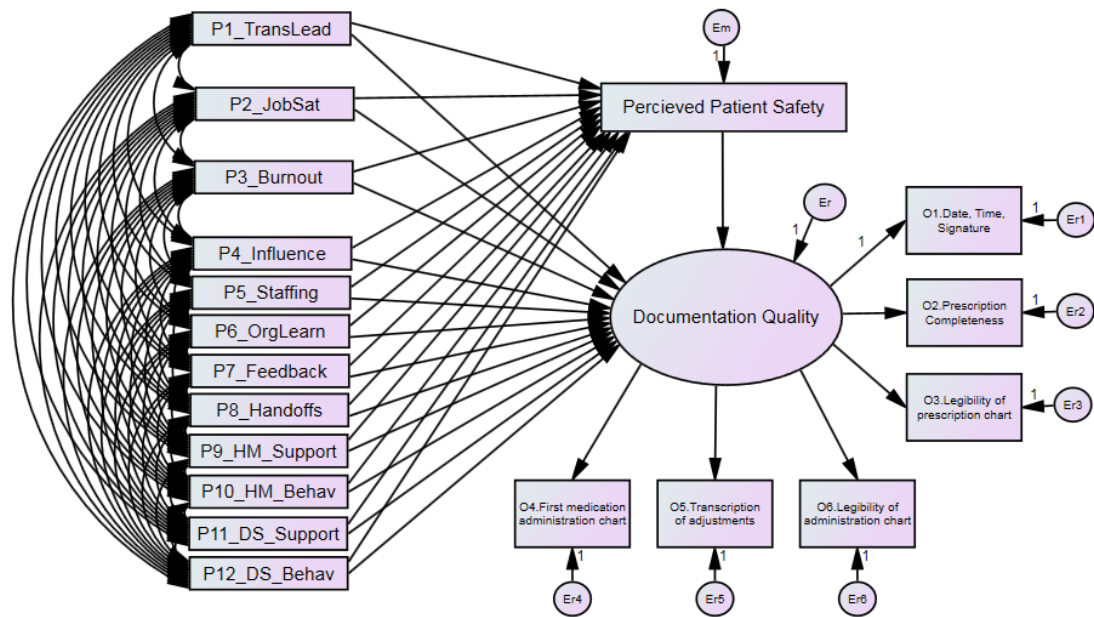

Supplement: S1 Appendix — The model used in the path analysis. (PDF) [file pone.0330499.s001.pdf]
