# Supplementary material for: A multi-group path analysis of medication documentation quality using cross-sectional survey data: Impact of leadership, job satisfaction, patient-related burnout, and patient safety culture
Source: PLoS One. 2025 Sep 12;20(9):e0330499. doi: 10.1371/journal.pone.0330499 (PMC12431215; doi:10.1371/journal.pone.0330499)
Supplement: S2 Appendix — The fit of the unconstrained multi-group model used in the path analysis. (PDF) [file pone.0330499.s002.pdf]

Supplementary Materials

Table S2: The fit of the unconstrained multi-group model used in the path analysis.

Model Fit Indices	Index benchmark (N>250 and 12<m<30)	Results of the analysis
Chi^2		806.7
df		148
p		<0.001
Chi^2/df	< 2.50	5.45
RMSEA	< 0.07	0.053
GFI	> 0.90	0.951
AGFI	> 0.90	0.873
NFI	≥ 0.95	0.972
NNFI	≥ 0.92	0.947
CFI	≥ 0.92	0.977

Note: Unconstrained Multi-group path analysis; N=802 for both analyses involving survey data from physicians and nurses in 24 departments.
