# Supplementary material for: A multi-group path analysis of medication documentation quality using cross-sectional survey data: Impact of leadership, job satisfaction, patient-related burnout, and patient safety culture
Source: PLoS One. 2025 Sep 12;20(9):e0330499. doi: 10.1371/journal.pone.0330499 (PMC12431215; doi:10.1371/journal.pone.0330499)
Supplement: Attachment — Submitted filename: WSM-PLOS ONE_Responce to reviewers.docx [file pone.0330499.s003.docx]

**Dear Editors and Reviewers,**

We sincerely appreciate the time and effort you have taken to evaluate our manuscript. We are grateful for the constructive feedback, which has helped us refine and improve our work. Below, we provide a detailed, point-by-point response to all comments.

We hope that our revisions have adequately addressed all concerns and that the improved manuscript meets the expectations of the journal. We thank you again for your thoughtful review and look forward to your feedback.

**Comments from the editorial office**

When submitting your revision, we need you to address these additional requirements.

1. Please ensure that your manuscript meets PLOS ONE's style requirements, including those for file naming. The PLOS ONE style templates can be found at

https://journals.plos.org/plosone/s/file?id=wjVg/PLOSOne_formatting_sample_main_body.pdf and

https://journals.plos.org/plosone/s/file?id=ba62/PLOSOne_formatting_sample_title_authors_affiliations.pdf

**Response:** Thank you for highlighting the importance of adhering to PLOS ONE's style requirements. We have thoroughly reviewed the guidelines provided by the journal and have adjusted our manuscript and file names accordingly to ensure full compliance. We hope you will find the revised documents meeting the set requirements.

2. Thank you for stating the following financial disclosure:

The „WorkSafeMed“ study was funded by the Federal Ministry of Education and Research (FKZ 01GY1325A and 01GY1325B). In addition, the work of the Institute of Occupational and Social Medicine and Health Services Research Tuebingen is supported by an unrestricted grant of the Employers´ Association of the Metal and Electric Industry Baden-Wuerttemberg (Suedwestmetall). We acknowledge additional financial support by the German Research Foundation and the Open Access Publishing Fund of the University of Tuebingen, and the administrative support by the DLR Project Management Agency.

Please state what role the funders took in the study. If the funders had no role, please state: "The funders had no role in study design, data collection and analysis, decision to publish, or preparation of the manuscript."

If this statement is not correct you must amend it as needed.

Please include this amended Role of Funder statement in your cover letter; we will change the online submission form on your behalf.

**Response:** We confirm that the funders had no role in the study design, data collection and analysis, decision to publish, or preparation of the manuscript. This statement will be included in our cover letter, as requested.

3. Your ethics statement should only appear in the Methods section of your manuscript. If your ethics statement is written in any section besides the Methods, please delete it from any other section.

**Response:** We have revised the manuscript and confirm that the ethics statement now exclusively appears in the Methods section.

4. In the online submission form, you indicated that due to data security and privacy regulations, data from the WorkSafeMed study cannot be shared publicly. However, data will be securely stored in compliance with national and regional data protection standards. Researchers who meet the criteria for access to confidential data may request access from AH, subject to reasonable conditions.

All PLOS journals now require all data underlying the findings described in their manuscript to be freely available to other researchers, either 1. In a public repository, 2. Within the manuscript itself, or 3. Uploaded as supplementary information.

This policy applies to all data except where public deposition would breach compliance with the protocol approved by your research ethics board. If your data cannot be made publicly available for ethical or legal reasons (e.g., public availability would compromise patient privacy), please explain your reasons on resubmission and your exemption request will be escalated for approval.

**Response:** We understand and support the journal's commitment to ensuring that data underlying the findings are accessible to enhance transparency and reproducibility. In the case of the WorkSafeMed study, the data involves sensitive personal information that is strictly governed by national and regional data protection laws. This dataset encompasses survey responses from clinical personnel and the reviews of the records of individual patients. Public deposition of this data would violate the ethical approval provided by our research ethics board, which mandates stringent measures to protect participant privacy and ensure compliance with legal requirements. A copy and English translation of the ethical approval is among the submission documents.

Consequently, while we cannot deposit the data in a public repository or include it directly within the manuscript or as supplementary information, we are committed to making the data available to researchers who meet stringent criteria for access to confidential data. This process will be managed through a formal data access request to the Institute for Patient Safety, Bonn (ifps@ukbonn.de), which will ensure any data sharing complies with the necessary confidentiality and ethical standards.

We have revised the Manuscript (declarations, availability of data and materials) and provided our rationale for this exemption and the mechanisms for data access. We kindly request your consideration and approval in accordance with the journal's policy for such exceptions.

5. We note that you have indicated that there are restrictions to data sharing for this study. For studies involving human research participant data or other sensitive data, we encourage authors to share de-identified or anonymized data. However, when data cannot be publicly shared for ethical reasons, we allow authors to make their data sets available upon request. For information on unacceptable data access restrictions, please see http://journals.plos.org/plosone/s/data-availability#loc-unacceptable-data-access-restrictions.

Before we proceed with your manuscript, please address the following prompts:

a) If there are ethical or legal restrictions on sharing a de-identified data set, please explain them in detail (e.g., data contain potentially identifying or sensitive patient information, data are owned by a third-party organization, etc.) and who has imposed them (e.g., a Research Ethics Committee or Institutional Review Board, etc.). Please also provide contact information for a data access committee, ethics committee, or other institutional body to which data requests may be sent.

b) If there are no restrictions, please upload the minimal anonymized data set necessary to replicate your study findings to a stable, public repository and provide us with the relevant URLs, DOIs, or accession numbers. Please see http://www.bmj.com/content/340/bmj.c181.long for guidelines on how to de-identify and prepare clinical data for publication. For a list of recommended repositories, please see https://journals.plos.org/plosone/s/recommended-repositories. You also have the option of uploading the data as Supporting Information files, but we would recommend depositing data directly to a data repository if possible.

Please update your Data Availability statement in the submission form accordingly.

**Response:** Please see the response to the previous comment for detailed discussion on data availability. The constraints imposed by the ethics committee are due to the sensitive nature of the data, prevent us from publicly sharing the data. We have updated the "Availability of Data and Materials" section of our manuscript to reflect these restrictions, indicating process for accessing the data upon reasonable request.

6. Your abstract cannot contain citations. Please only include citations in the body text of the manuscript, and ensure that they remain in ascending numerical order on first mention.

**Response:** We have reviewed the abstract, ensuring that it does not include any citations. We have also reviewed the entire manuscript to confirm that citations in the body text are in ascending numerical order on first mention.

**Reviewers' comments:**

Reviewer's Responses to Questions

Comments to the Author

1. Is the manuscript technically sound, and do the data support the conclusions?

The manuscript must describe a technically sound piece of scientific research with data that supports the conclusions. Experiments must have been conducted rigorously, with appropriate controls, replication, and sample sizes. The conclusions must be drawn appropriately based on the data presented.

Reviewer #1: Yes

Reviewer #2: Yes

Reviewer #3: Yes

2. Has the statistical analysis been performed appropriately and rigorously?

Reviewer #1: Yes

Reviewer #2: Yes

Reviewer #3: Yes

3. Have the authors made all data underlying the findings in their manuscript fully available?

The PLOS Data policy requires authors to make all data underlying the findings described in their manuscript fully available without restriction, with rare exception (please refer to the Data Availability Statement in the manuscript PDF file). The data should be provided as part of the manuscript or its supporting information, or deposited to a public repository. For example, in addition to summary statistics, the data points behind means, medians and variance measures should be available. If there are restrictions on publicly sharing data—e.g. participant privacy or use of data from a third party—those must be specified.

Reviewer #1: Yes

Reviewer #2: Yes

Reviewer #3: Yes

4. Is the manuscript presented in an intelligible fashion and written in standard English?

PLOS ONE does not copyedit accepted manuscripts, so the language in submitted articles must be clear, correct, and unambiguous. Any typographical or grammatical errors should be corrected at revision, so please note any specific errors here.

Reviewer #1: Yes

Reviewer #2: Yes

Reviewer #3: Yes

**5. Review Comments to the Author**

Please use the space provided to explain your answers to the questions above. You may also include additional comments for the author, including concerns about dual publication, research ethics, or publication ethics. (Please upload your review as an attachment if it exceeds 20,000 characters)

**Reviewer #1:**

Thank you for submitting your study to our journal.

Your study addresses a crucial issue in healthcare—medication documentation quality and its organizational predictors—which is an essential contribution to patient safety literature. The methodology is robust, using validated tools and multi-group path analysis, and the dataset is comprehensive and valuable. To further enhance your manuscript, I have some suggestions for improvement.

**Response:** We appreciate your encouraging evaluation, as well as careful recommendations to improve the manuscript. Below we address each of them individually.

5.1.1. There are minor grammatical errors, such as "To what extend," which should be "extent." A thorough review of the text is recommended to address these issues.

**Response:** Thank you for pointing out the minor grammatical issues present in our manuscript. We have conducted a thorough review of the text to correct these issues, including the specific error you noted. We hope you will find that the revisions have improved the overall clarity and readability of our manuscript.

5.1.2. While the sample size is sufficient, the 39.6% response rate may introduce response bias. It would be helpful to discuss how this might affect the generalizability of your findings.

**Response:** We agree and acknowledge that while our sample size is sufficient for analysis, the response bias can not be excluded, which might limit the generalizability of our findings. In response, we have extended the discussion section to address this issue explicitly.

5.1.3. Aggregating survey data at the department level could dilute individual-level variations. Please provide a clear justification for why this approach was chosen.

**Response:** Thank you for raising the issue of the data aggregation. In our study, we utilized two primary data sources: surveys, conducted at the individual employee level, and record reviews, at the individual patient level. Due to the nature of these data sets, the most viable option to match and analyze these simultaneously was to aggregate the data at the department level. This approach is justified as the constructs evaluated in the employee survey, such as safety culture or job satisfaction, are considered to be group-level constructs, typically analyzed at the department or even hospital level in health services research.

We recognize the importance of clarifying this methodological decision and have revised the methods section to make this rationale more transparent.

5.1.4. The negative perceived patient safety 'mediation effect' observed for nurses may require further exploration.

**Response:** We appreciate you highlighting this interesting and unexpected mediation effect. The negative relationship between Perceived Patient Safety and Documentation Quality for nurses remains an open question that requires further investigation. While we have provided some possible explanations—such as overconfidence in highly safe environments or the impact of workload limiting documentation time—the underlying mechanisms are not yet fully understood. It is also possible that additional factors not captured in our study contribute to this effect. We have expanded the Discussion section to acknowledge these possibilities and emphasize the need for further research to explore this complex relationship.

5.1.5. The positive job satisfaction for nurses but not physicians highlights professional differences, which may require more clarification and may help in tailored intervention.

**Response:** Thank you for highlighting the significant effect of job satisfaction for nurses but not physicians. That this point is clearly underlined in the revised discussion.

5.1.6. While the theoretical model is sound, it needs to fully explain the variance in outcomes. Consider refining the model by including additional predictors like workload, team dynamics, or individual factors like years of experience.

**Response:** We appreciate your suggestion to enhance our theoretical model by including additional predictors. While we agree that this could enrich our findings, we are constrained by the original study design and the data currently available to us. We have extended the limitation section with a recommendation for future studies to include additional factors, which may extend our understanding of the complex interplay between organizational factors and the quality of medical documentation.

5.1.7. While the ethics statement is thorough, the decision to restrict data availability does not align with PLOS ONE's data-sharing policy. Consider providing anonymized data or offering a more precise justification for the restrictions.

**Response:** Thank you for your feedback on data availability. As noted in our response to the Editor's Comment #4, strict constraints from the ethical approval prevent sharing data publicly. We have detailed these constrains and the procedure for obtaining the data in the Data Availability statement accordingly.

**Reviewer #2:**

The study, titled "A multi-group path analysis of medication documentation quality using cross-sectional survey data: Impact of leadership, job satisfaction, patient-related burnout, and patient safety culture," examines a crucial area of healthcare: the quality of medication documentation and its relationship with organizational and individual factors. The research provides a significant contribution to the understanding of how leadership, job satisfaction, burnout, and safety culture influence documentation practices, which are directly linked to patient safety and the prevention of adverse medication events.

The methodological framework is robust, employing a multi-group path analysis to explore the interplay between organizational factors and documentation quality. By using data collected from 24 departments across two German university hospitals, the study incorporates survey responses from frontline workers—282 physicians and 417 nurses—and retrospective reviews of 802 patient records. This dual approach allows for a comprehensive analysis that links staff perceptions to objective outcomes in documentation quality. Established instruments, such as the Transformational Leadership Inventory and the Copenhagen Psychosocial and Burnout Inventories, lend credibility to the survey data, while the MediDoq instrument ensures a standardized and detailed assessment of medication documentation.

One of the study's strengths is its focus on comparing the effects of the examined factors across professional groups. The findings reveal significant differences between physicians and nurses, particularly in how perceived patient safety mediates the relationship between organizational factors and documentation quality. For nurses, perceived patient safety played a substantial mediating role, while for physicians, it did not. These nuanced findings highlight the importance of tailoring interventions to the specific needs and dynamics of professional groups within healthcare settings.

Despite its strengths, the study has some limitations. The high level of aggregation of survey data at the department level may obscure individual-level variations and introduce potential biases.

Additionally, the high response rate for surveys (approximately 39.6%) is commendable, but the lack of long-term follow-up limits insights into sustained impacts. Furthermore, while the multi-group path analysis demonstrates a good fit with the data, the study acknowledges that other unexplored factors may influence documentation quality, leaving room for future research to build on these findings.

In conclusion, this study provides a valuable framework for understanding the complex relationships between organizational factors and medication documentation quality. Its methodological rigor and practical implications make it a strong candidate for publication. With its findings, the study not only advances academic understanding of patient safety culture but also offers actionable insights for improving healthcare practices. Nonetheless, addressing the stated limitations and expanding the scope of analysis in future research would further enhance the robustness and applicability of the findings.

**Response:** Thank you for your thorough and constructive review of our manuscript. We are grateful for your positive evaluation of the methodological rigor and comprehensive analysis presented in our study, as well as for acknowledging the practical implications of our findings.

We appreciate your insights regarding the limitations of data aggregation and the cross-sectional design of our study. We acknowledge these as inherent constraints and have expanded the limitations section of our manuscript to address these concerns more comprehensively. Additionally, we have noted the importance of incorporating longitudinal designs in future research to better explore causal relationships and the sustained impacts of the factors on documentation quality.

**Reviewer #3:**

GENERAL COMMENTS

5.3.1. The paper is well-written and encounters a very important topic. Some editing points need to be checked about grammar and punctuation.

**Response:** Thank you for acknowledging the importance of the topic of the manuscript, and for your positive feedback on its overall composition. We appreciate your suggestion to improve the grammar and punctuation. We have thoroughly reviewed the manuscript and performed additional editing to ensure clarity and correctness in these areas.

DETAILED COMMENTS:

INTRODUCTION:

5.3.2. It covers the background literature and study aims and objectives. However, it would be informative to document any previous similar study predicting individual or organizational factors affecting the quality of medication documentation. In addition, it is important to highlight at the end of the introduction why you are conducting the current study (e.g. lack of literature, what this study will add or fulfill the gaps in the literature).

**Response:** Thank you for your constructive feedback regarding the introduction of our manuscript. We have clarified the introduction to highlight the limited evidence on factors affecting medication documentation quality and expanded on why our study, focusing on organizational factors, is needed.

5.3.3. Page 3, line 46: "A recent metanalysis found, that for hospital settings, as high as 19% of all hospitalized patients…." When checking the reference used (Ref 1), the paper was published in 2017 which is not recent. I suggest you either update the ref with a more recent meta-analysis or paraphrase the sentence. Please remove the comma after "found."

**Response:** Thank you for pointing out this oversight. After reviewing the latest available literature, we found no newer meta-analyses on the topic. Therefore, we have adjusted the sentence, and corrected the grammatical error by removing the comma following 'found'.

5.3.4. Page 3, line 69: Please check the grammar; the word "effects" should be changed to the verb "affects".

**Response:** We have revised the sentence accordingly.

5.3.5. Page 4, lines 78-79: Please move ref (10) to the end of the sentence.

**Response:** We have revised the sentence accordingly.

5.3.6. Page 4, lines 86-87: Please change " The model, developed based on………, is presented on figure 1" to the model, developed based on………, is presented in figure 1"

**Response:** We have revised the sentence accordingly.

METHODS

In the methods section, please clarify the following:

5.3.7. How the conceptual framework was developed. Were there any validation steps undertaken for the framework?

**Response:** Thank you for your question. The conceptual framework was developed based on theoretical considerations and prior research. While we did not conduct a formal external validation of the framework, we assessed the fit indices to evaluate whether the theoretical model aligned with the data, supporting its plausibility. Future research could extend this work by validating the framework in different clinical settings or through longitudinal studies.

5.3.8. For the MediDocQ Instrument, not all the original criteria described in ref 7 were used in this study. So how this will affect the analysis and interpretation of results?

**Response:** Thank you for your comment. We acknowledge that our study uses a subset of the MediDocQ criteria. The selection of the items from the MediDocQ instrument was primarily based on data availability across patient records, as well as the relevance of specific criteria to our research objectives. While this may limit comparability with other studies using the full MediDocQ set, the included criteria still capture key aspects of medication documentation quality. We made sure, that all selected criteria are transparently presented in the manuscript, ensuring clarity in the analysis and interpretation of results. Given this transparency, we do not anticipate any issues in analyses or interpretation of our findings.

5.3.9. Did you carry out any sample size calculations?

**Response:** Thank you for your comment. While no formal sample size calculation was conducted, we ensured an appropriate sample size for multivariate analyses by including inpatient units treating at least 500 patients per year. We revised the Methods section to clarify this.

5.3.10. Please clarify the sampling strategy. The HCPs were selected by convenience as mentioned in the manuscript, however, how were the departments selected?

**Response:** Thank you for your question. We have clarified the department selection process in the Methods section. All inpatient units treating at least 500 patients per year were included to ensure sufficient documentation data, while intensive care and psychiatric units were excluded due to their distinct documentation practices.

5.3.11. Please write the inclusion/exclusion criteria in the methods section.

**Response:** According to your suggestion, we have revised Methods section to include the inclusion and exclusion criteria for departments.

RESULTS:

5.3.12. Well presented. However, one point is about table 1 (page 9): the results from HSPSC were expressed as mean (+/-SD), although the AHRQ's guide for survey analysis is to express the results by positive response rate. How would this affect the interpretation of the results?

**Response:** Thank you for your insightful observation. We acknowledge that the AHRQ guide recommends expressing HSPSC results as positive response rates. However, we chose to report the results as mean (± SD) to maintain consistency with the other surveyed variables included in our path model. While positive response rates are useful for benchmarking, our focus was on the relative differences across professional groups and their associations with Documentation Quality. We recognize this as a methodological choice and have added a clarification in the manuscript to ensure transparency in interpretation.

DISCUSSION

5.3.13. Page 14, line 314: the net difference of 1.03 and -1.21 is -0.18 not -0.19. please correct.

**Response:** Thank you for your careful attention to detail. We have rechecked the calculations and confirm that the reported values are correct as extracted from the statistical software. The apparent discrepancy is due to rounding. To clarify this, we have added a note to the table indicating that slight differences may result from rounding.

5.3.14. Page 14, line 320: Please insert ref after the first sentence.

**Response:** Thank you for your suggestion. We have added the appropriate reference.

5.3.15. Page 14, line 331: Please change the sentence "…..should be considered when planning research or improvement projects,…" to sentence "…..should be considered when planning research or improving projects,…"

**Response:** Thank you for your suggestion. The sentence has been revised as part of the general grammar revision to improve clarity.

5.3.16. What are the implications of this study on the clinical practice? Please indicate in the manuscript.

**Response:** Thank you for your suggestion. We have added a separate section on "Practical Implications" in the Discussion, highlighting the relevance of organizational factors and the need for tailored interventions to enhance documentation quality and patient safety.
